# Resting‐state functional connectivity of the occipital cortex in different subtypes of Parkinson's disease

**DOI:** 10.1111/cns.14915

**Published:** 2024-08-26

**Authors:** Yina Lan, Hongjun Yuan, Xiaoxaio Ma, ChunYu Yin, Xinyun Liu, XiYu Zeng, Jinhao Lyu, Yongqin Xiong, Xiaobo Zhang, Haoxuan Lu, Yujue Zhong, Xuemei Li, Zhiqiang Cui, Xin Lou

**Affiliations:** ^1^ Department of Radiology The First Medical Center of Chinese PLA General Hospital Beijing China; ^2^ Department of Radiology The Fifth Medical Center of Chinese PLA General Hospital Beijing China; ^3^ Department of Cadres' Outpatient The First Medical Center of Chinese PLA General Hospital Beijing China; ^4^ Department of Neurosurgery The First Medical Center of Chinese PLA General Hospital Beijing China

**Keywords:** functional connectivity, motor subtype, Parkinson's disease

## Abstract

**Aims:**

To examine whether functional connectivity (FC) of the occipital gyrus differs between patients with Parkinson's disease (PD) motor subtypes and healthy controls (HCs).

**Methods:**

We enrolled 30 PD patients exhibiting tremor dominance (TD), 43 PD patients with postural instability and gait disturbance (PIGD), and 42 HCs. The occipital gyrus was partitioned into six areas of interest, as seed points, via the Anatomical Automatic Labeling template to compare the FC of the three groups and analyze the relationship of FC with clinical scales.

**Results:**

Compared with the PIGD group, the TD group showed increased FC between the left superior occipital gyrus (SOG.L) and right median cingulate and paracingulate gyri (DCG.R)/right paracentral lobule/bilateral inferior parietal, but supramarginal and angular gyri; the left middle occipital gyrus (MOG.L) and left posterior cingulate gyrus (PCG.L); the MOG.R and SOG.L/right calcarine fissure and surrounding cortex/DCG.R/PCG.L/right cuneus; the left inferior occipital gyrus (IOG.L) and right caudate nucleus; and the IOG.R and PCG.L.

**Conclusion:**

Differentiated FC between the occipital gyrus and other brain areas within the PD motor subtypes, which may serve as neural markers to distinguish between patients with TD and PIGD PD.

## Graphical abstract

The differentiated FC between the occipital gyrus and other brain areas within the PD motor subtypes, which may serve as neural markers to distinguish between patients with tremor dominance and postural instability and gait disturbance Parkinson's disease.

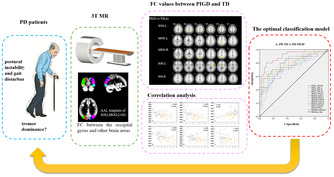

## 1 INTRODUCTION

More than 6 million people live with Parkinson's disease (PD) worldwide, making it the second most common neurodegenerative disease.^1^ The clinical manifestations of PD vary according to motor subtype. Common classifications include tremor dominance (TD), intermediate, and postural instability and gait disturbance (PIGD).^2^ In contrast to the TD subtype, the PIGD subtype progresses rapidly and has a poor prognosis.^3^ However, the neuropathological mechanisms underlying the differences between the two motor subtypes of PD remain unclear. The occipital region can regulate gait under visual guidance,^4^ and movements of patients with PD are increasingly controlled by vision.^5^ Magnetic resonance imaging (MRI) research has indicated that motor impairment is related to the occipital region in PD patients. PD patients have larger white matter (WM) hyperintensity in the occipital region compared to healthy controls (HCs), accompanied by deteriorated motor signs.^6^ The fractional amplitude of low‐frequency fluctuation (fALFF) values in the occipital gyrus of PD patients increased after 2 years, compared with the baseline.^7^

Furthermore, other MRI studies have demonstrated differences in the occipital region between the TD and PIGD motor subtypes. For instance, in the PIGD subgroup, functional connectivity (FC) between the STN and bilateral middle occipital gyrus (MOG) was greater, which positively correlated with PIGD scores in patients with PD.^8^ Patients with TD had higher FC between the MOG.L and thalamic subregions than patients with PIGD.^9^ PD tremor improvement was related to reduced fALFF values in the left occipital cortex (BA17).^10^ PD patients with tremors had reduced gray matter (GM) volumes in the MOG.L.^11^ Tremor severity correlated with the loss of volume in the left occipital lobe.^12^ PIGD PD had higher ALFF values in the MOG.L and left superior occipital gyrus (SOG.L) than TD PD.^13^ Based on these studies, the motor manifestations of PD are closely related to the occipital region.

Nevertheless, none of these studies used the occipital gyrus as seed points to explore the differences in FC between the two motor subtypes. In recent years, resting‐state functional magnetic resonance imaging (R‐fMRI) has become a common tool for studying FC in the motor subtypes of PD.^8^, ^9^, ^14^ Therefore, we applied the Anatomical Automatic Labeling (AAL) template to divide the occipital gyrus into six functional subregions: bilateral SOG, bilateral MOG, and bilateral inferior occipital gyrus (IOG). We hypothesized that FC differences would exist between the six subregions of the occipital gyrus and other brain areas among different PD motor subtypes. Different motor subtypes of PD can be distinguished based on these differences, which can help us understand their neuropathological mechanisms.

## 2 METHODS

### 2.1 Participants

This retrospective study was approved by the institutional review board (reference number: S2022‐572‐01), which waived the necessity for written informed consent. We enrolled 30 TD PD patients, 43 PIGD PD patients, and 42 HCs. The research protocol was as follows. The following requirements were applied to all participants: (1) right‐handedness, (2) a signed informed consent form, (3) age >40 years, and (4) the ability to undergo MRI examinations. PD was diagnosed by two neurologists with >10 years of experience. PD patients were included based on the following criteria: (1) normal Mini‐Mental State Examination (MMSE) scores (illiterate participants scored >17, grade‐school‐educated participants scored >20, junior high school, and higher‐education‐literate higher‐educated participants scored >23),^15^ and (2) disease duration >5 years. Following were the criteria for excluding PD patients: (1) intermediate motor subtype; (2) severe depression, anxiety, schizophrenia, and other mental illnesses; and (3) poor image quality or large head‐motion artifacts. We collected demographic and clinical data, including sex, age, years of education, GM volume, disease duration, and daily levodopa dosage.

The clinical scales were assessed before the MRI scan, and medication was discontinued at least 12 h before the scan. Clinical scales included the following: Movement Disorder Society‐Sponsored Revision of the Unified PD Rating Scale (MDS‐UPDRS), Berg Balance Scale (BBS), Hoehn and Yahr stage, Argentina Hyposmia Rating Scale (AHRS), Hamilton Anxiety Scale (HAMA), MMSE, Epworth Sleepiness Scale (ESS), Hamilton Depression Scale (HAMD), and Rapid‐eye‐movement Sleep Behavior Disorder Questionnaire Hongkong (RBDQ‐HK). PD subtypes were classified using MDS‐UPDRS, by the ratio of the mean tremor scores (MDS‐UPDRS II item 2.10 and MDS‐UPDRS III items 3.15–3.18)/11 to the mean PIGD scores (MDS‐UPDRS II item 2.12–2.13 and MDS‐UPDRS III items 3.10–3.12)/5, and the participants were classified as patients with TD (ratio ≥1.15 or PIGD score = 0 and TD score >0), patients with PIGD (ratio ≤0.9 and PIGD score ≠ 0), and patients with indeterminate PD (0.9<ratio<1.15 or both TD and PIGD scores = 0).^16^

### 2.2 Image acquisition

MRI data were collected using a 3.0‐T scanner (GE Discovery MR750, Milwaukee, WI, USA). During MRI acquisition, participants were instructed to close their eyes, remain relaxed, and refrain from napping or engaging in deliberate thought. Foam padding and earplugs were used to reduce noise and head movements. The parameters of R‐fMRI and three‐dimensional fast spoiled gradient recalled (3DFSPGR) were consistent with our previous research.^13^ The R‐fMRI data were pre‐processed using the Statistical Parametric Mapping (SPM) 12 toolbox in MATLAB 2022b and the REST version 1.2 (http://www.restfmri.net). In general, the following steps were as follows: (1) removing the first 10 time points, (2) making corrections to the remaining images based on slice timing, (3) correction of head movement (head shift or angular rotation >2), (4) co‐registration of the functional images with the T1‐weighted images and spatial normalization, (5) spatial smoothing using a full‐width half‐maximum (FWHM) Gaussian kernel of 6 mm, (6) eliminating the linear trend, (7) regression analysis, and (8) temporal band‐pass filtering (0.01–0.08 Hz).^17^ Six subregions of the occipital region of the AAL, including the bilateral SOG, bilateral MOG, and bilateral IOG, were adopted for this study. FC analysis was conducted by calculating the temporal Pearson's correlations between the average time series of each region of interest (ROI) and the time series of each voxel in the brain. Using Fisher's r‐to‐z transformation, a *z*‐score map was created for each ROI per participant based on the resulting connectivity maps. The 3DFSPGR data were pre‐processed using the SPM8 toolbox in MATLAB 2013b. Following are the steps involved in data processing: (1) spatial normalization; (2) division of the normalized image into GM, WM, and cerebrospinal fluid signal; and (3) spatial smoothing (FWHM Gaussian kernel = 8 mm).

### 2.3 Statistical analysis

Demographic and clinical data were analyzed using IBM SPSS (v. 23.0, Armonk, NY, USA). Statistical significance was set at *p* < 0.05. Quantitative variables were checked for normality by the Kolmogorov–Smirnov test. Sex was compared using chi‐square tests, whereas the Kruskal–Wallis test was used to compare age and GM volume for TD PD, PIGD PD, and HCs. Years of education, duration of illness, daily levodopa dosage, MDS‐UPDRS, Hoehn and Yahr stage, AHRS, MMSE, HAMD, HAMA, tremor score, ESS, PIGD score, BBS, and RBDQ‐HK scores were analyzed using the Mann–Whitney *U* test. Age, GM volume, and sex were used as covariates in the analysis of covariance to compare the FC values among the three groups. A two‐sample *t*‐test was used for post‐hoc analysis. Additionally, we used multiple linear regression analyses to investigate the relationship between FC values and clinical scores within the mask showing differences among the three groups, with age and sex as covariates for patients with TD and PIGD PD, and HCs. The statistical significance threshold was set at uncorrected voxel‐wise (*p* < 0.001) and family‐wise error‐corrected (FWE) cluster‐wise (*p* < 0.05) values. Data analysis was performed using the SPM12. We used IBM SPSS (v. 23.0; Armonk, NY, USA) to analyze the predictive value of each FC index among the TD, PIGD, and HC groups based on the receiver operating characteristic curve (ROC). Subsequently, a binary logistic regression model was built, and the predicted probability value of the logistic regression was used as a new comprehensive indicator to continue the ROC curve. Statistical significance was set at *p* < 0.05. We calculated the ROC and area under the curve (AUC) to evaluate the sensitivity and specificity of single and multivariate indicators.

## 3 RESULTS

### 3.1 Demographic and clinical data

We enrolled 115 participants in this study, including 73 patients with PD (30 with TD/43 with PIGD) and 42 HCs. The clinical and demographic data of the participants are summarized in Table 1.

**TABLE 1:** Demographic and clinical data of all the subjects.

	TD (*n* = 30)	PIGD (*n* = 43)	HC (*n* = 42)	*p*
Gender	9:21	20:23	22:20	0.159
Age	62.20 ± 9.67	64.16 ± 7.08	61.40 ± 7.14	0.131
GM volume	607.03 ± 41.29	590.41 ± 55.30	616.66 ± 49.36	0.109
Disease duration	7.63 ± 5.55	8.84 ± 4.98	–	0.107
Education (year)	10.43 ± 4.50	11.30 ± 3.26	–	0.487
H‐Y stage	2.33 ± 0.42	3.05 ± 0.60	–	0.000
UPDRSI	9.20 ± 5.28	13.30 ± 5.87	–	0.004
UPDRSII	12.23 ± 5.48	21.58 ± 7.91	–	0.000
UPDRSIII	51.00 ± 16.43	52.42 ± 13.919	–	0.715
UPDRSIV	4.37 ± 3.41	6.93 ± 3.97	–	0.007
Levodopa daily dosage (mg/day)	467.50 ± 275.833	553.49 ± 312.37	–	0.213
Tremor score	1.59 ± 0.59	0.47 ± 0.47	–	0.000
PIGD score	0.73 ± 0.43	1.88 ± 0.64	–	0.000
AHRS	19.30 ± 5.88	18.23 ± 6.73	–	0.641
MMSE	26.50 ± 2.69	24.74 ± 6.23	–	0.355
HAMA	5.50 ± 5.00	7.05 ± 5.07	–	0.136
HAMD	6.77 ± 5.04	9.47 ± 6.87	–	0.066
BBS	49.53 ± 6.70	39.42 ± 12.57	–	0.000
ESS	4.13 ± 4.02	7.33 ± 5.46	–	0.013
RBDQ‐HK	17.33 ± 15.50	22.65 ± 16.1		0.149

### 3.2 FC values in the TD, PIGD, and HCs

**FIGURE 1:**
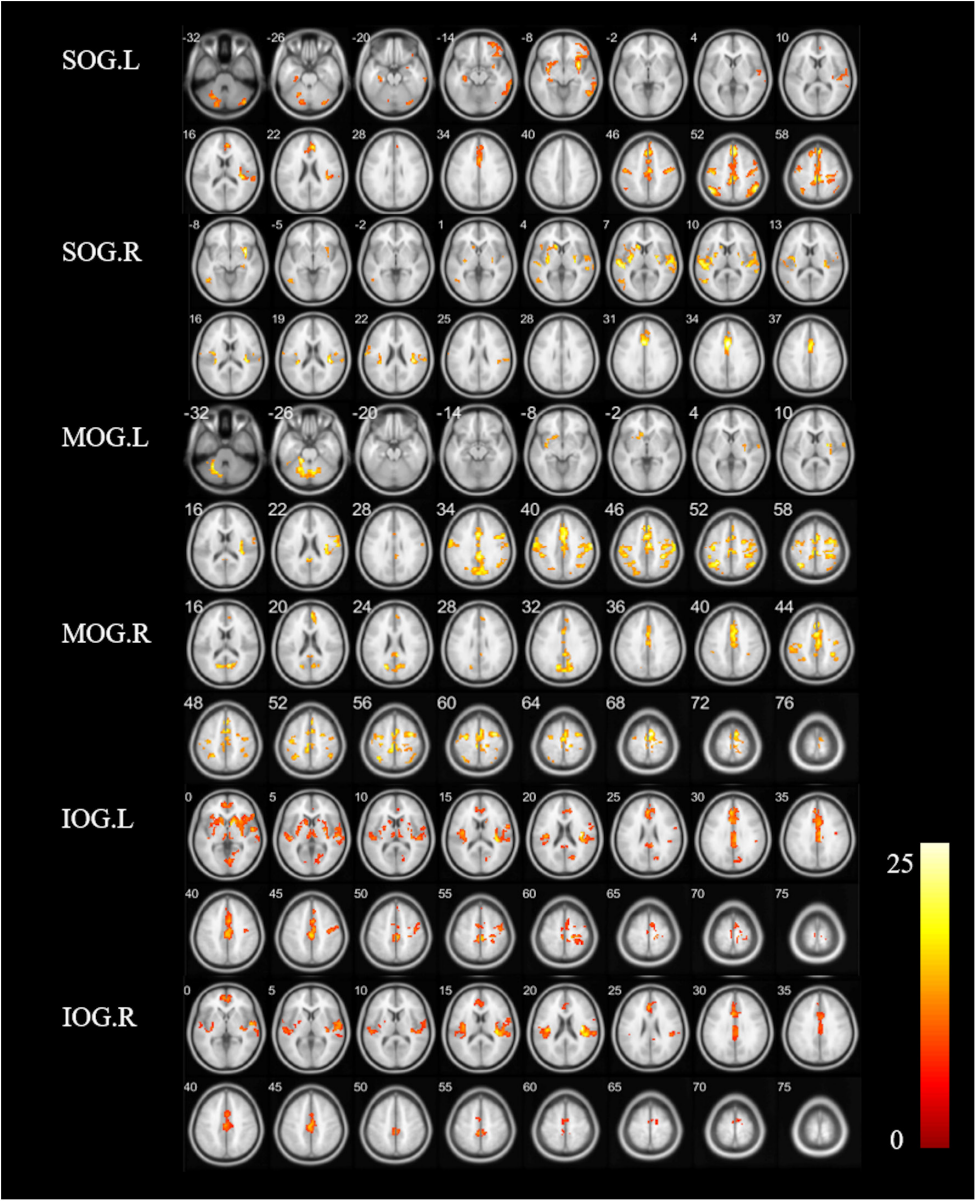
Results of significant difference in FC values among the TD, PIGD, and HCs. HCs, healthy controls; IOG, inferior occipital gyrus; MOG, middle occipital gyrus; PIGD, postural instability, and gait dysfunction; SOG, superior occipital gyrus; TD, tremor dominant.

The FC values among patients with TD and PIGD PD and HCs (cluster *p* < 0.05, FWE corrected) are shown in Figure 1 and Table 2. The following shows the specific FC of the subregions in the occipital gyrus cluster (*p* < 0.05, FWE corrected).

**TABLE 2:** Results of FC values among the TD, PIGD, and HCs.

Seed ROI	Brain regions (AAL)	Peak value			Voxels	*F* value
		*X*	*Y*	*Z*		
SOG.L	CERCRU1.L	−15	−75	−24	179	11.588
	CERCRU1.R	42	−75	−33	90	12.6049
	HIP.L	−33	−6	−9	132	12.9937
	ORBinf.R	33	6	−9	270	20.9518
	ITG.R	51	−54	−9	172	12.1172
	HES.R	36	−27	15	234	17.6196
	DCG.R	6	39	30	256	18.5412
	IPL.L	−42	−60	54	143	21.7536
	SFGmed.R	3	27	51	989	19.6985
	PoCG.L	−48	−12	48	129	16.5719
	SFGmed.L	−3	27	51	96	15.3877
	IPL.R	48	−54	54	149	22.5411
SOG.R	HIP.R	33	6	−9	139	17.1945
	MTG.L	−48	−63	9	87	11.0706
	INS.R	36	−27	18	326	17.2273
	STG.L	−51	−24	6	329	15.1268
	DCG.R	3	12	33	157	17.461
	SMA.R	3	−3	60	434	16.3116
	PreCG.L	−27	−12	60	96	12.334
MOG.L	CER6. L	−21	−60	−30	444	14.2777
	INS.L	−36	0	−6	87	10.2478
	INS.R	36	−15	21	108	14.1034
	PCL.L	−3	−15	69	2282	16.8541
	IPL.L	−30	−54	54	258	15.7413
	IPL.R	48	−54	54	173	12.6456
	PreCG.L	−33	−9	66	431	13.3546
MOG.R	CAL.R	6	−66	15	253	14.3175
	SMA.R	6	−3	69	1145	17.2475
	IPL.R	45	−51	54	114	12.6881
	PreCG.L	−27	−9	60	230	13.176
	SFGdor.R	33	−3	60	175	15.3568
	IPL.L	−33	−57	54	95	12.582
IOG.L	CER 4_5.L	−15	−39	−27	81	16.4651
	LING.R	6	−75	0	224	11.9369
	STG.L	−45	−24	3	406	15.3288
	INS.R	36	−15	21	713	25.8345
	ACG.L	−3	33	27	1208	16.3975
	THA.R	9	−21	3	99	10.8645
	PreCG.R	42	−15	48	165	12.2928
	PoCG.R	15	−30	60	139	15.9448
IOG.R	INS.R	36	−15	21	532	27.111
	ACG.L	−6	51	0	71	13.0321
	INS.L	−36	−21	21	355	17.3274
	DCG.L	−3	−21	45	696	15.8324
	SMA.L	−6	−3	57	87	11.1024

### 3.3 TD group versus PIGD group

**FIGURE 2:**
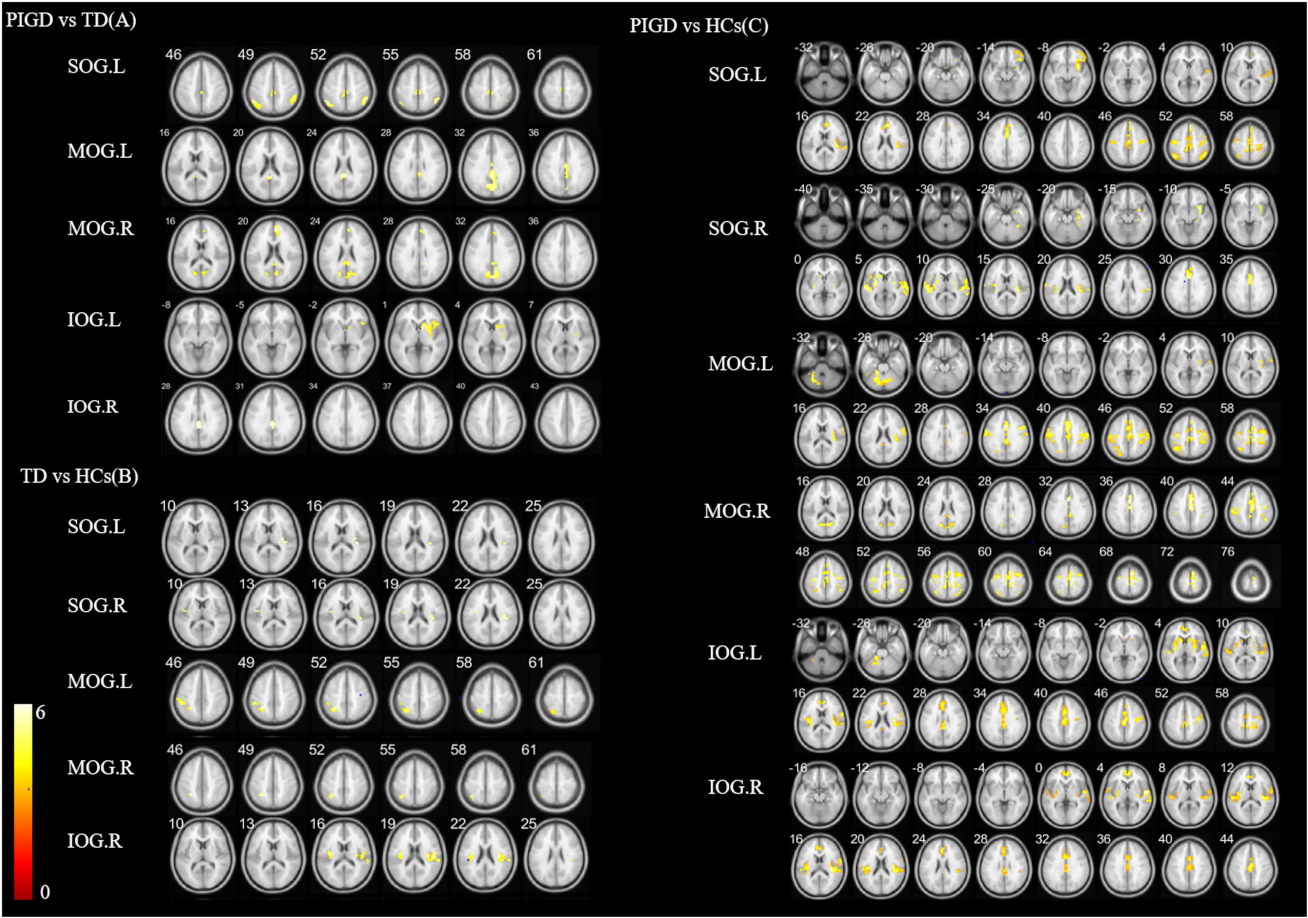
Results of the two‐sample *t*‐test of FC values between PIGD and TD (A); TD and HCs (B); PIGD and HCs (C).

Compared with the PIGD group, the TD group showed increased FC between the SOG.L and right median cingulate and paracingulate gyri (DCG.R)/right paracentral lobule (PCL.R)/bilateral inferior parietal, but supramarginal and angular gyri (IPL), between the MOG.L and left posterior cingulate gyrus (PCG.L), between the MOG.R and SOG.L/right calcarine fissure and surrounding cortex (CAL.R)/DCG.R/PCG.L/right cuneus (CUN.R), between the IOG.L and right caudate nucleus (CAU.R), and between the IOG.R and PCG.L (*p* < 0.05 FWE corrected, Figure 2A and Table 3).

**TABLE 3:** Results of the Two‐sample *t*‐test of FC values between groups.

Seed ROI	Brain regions (AAL)	Peak value			Voxels	*T* value
		*X*	*Y*	*Z*		
PIGD<TD						
SOG.L	DCG.R	9	39	30	39	−4.2915
	PCL.R	3	−33	51	76	−4.2561
	IPL.L	−48	−57	51	59	−4.79
	IPL.R	48	−51	54	68	−4.9503
MOG.L	PCG.L	−3	−45	18	225	−4.6624
MOG.R	SOG.L	−18	−69	24	52	−4.6553
	CAL.R	6	−66	18	71	−4.7237
	DCG.R	9	36	30	125	−5.0048
	PCG.L	−3	−45	18	53	−4.4371
	CUN.R	3	−78	33	65	−5.0792
IOG.L	CAU.R	9	9	0	110	−5.2098
IOG.R	PCG.L	0	−33	30	45	−3.8902
PIGD<HC						
SOG.L	ORBinf.R	33	6	−9	264	−6.6558
	HES.R	36	−27	15	233	−5.87
	ACG.L	0	12	30	256	−6.45
	IPL.L	−42	−60	54	135	−5.7497
	SFGmed.R	3	27	51	989	−6.366
	PoCG.L	−42	−18	51	129	−5.0452
	IPL.R	48	−54	54	141	−6.0171
SOG.R	HIP.R	33	6	−9	135	−5.7471
	PUT.R	30	−15	3	291	−5.5493
	STG.L	−54	−21	6	311	−5.3732
	SMG.R	63	−24	24	27	−4.3199
	DCG.R	3	12	33	157	−5.7447
MOG.L	CER 4_5.L	−18	−39	−27	442	−5.4275
	ROL.R	36	−24	18	108	−5.4818
	DCG.L	0	9	33	2126	−6.226
	SPG.L	−33	−57	57	240	−4.643
	PoCG.L	−51	−18	42	407	−4.9968
MOG.R	CAL.R	6	−66	15	180	−4.9474
	SMA.R	6	−3	69	1021	−5.7301
	IPL.R	45	−51	54	106	−4.6545
	PreCG.L	−27	−9	60	226	−4.7048
	SFGdor.R	33	−3	60	174	−4.7253
	SPG.L	−33	−57	57	83	−4.4964
IOG.L	CER 4_5.L	−15	−39	−27	80	−5.7347
	STG.L	−45	−24	3	388	−6.2088
	ROL.R	36	−21	18	639	−6.859
	DCG.L	0	−24	45	1195	−5.5754
	PreCG.R	42	−18	48	165	−4.9329
	PoCG.R	15	−30	60	139	−5.4132
IOG.R	ROL.R	36	−21	18	518	−6.9341
	SFGmed.L	−3	51	3	70	−5.3777
	ROL.L	−39	−21	21	347	−5.823
	DCG.L	0	−18	45	696	−5.736
TD<HC						
SOG.L	ROL.R	39	−30	15	41	−3.9604
SOG.R	ROL.L	−42	−9	12	21	−3.7497
	ROL.R	39	−27	21	44	−4.2183
MOG.L	IPL.L	−30	−51	48	120	−5.2544
MOG.R	IPL.L	−30	−51	48	54	−4.5187
IOG.R	INS.R	36	−18	21	124	−5.0721
	INS.L	−36	−12	15	60	−4.3237

### 3.4 TD group versus HC group

Compared with the HC group, the TD group showed decreased FC between the SOG.L and right rolandic operculum (ROL.R), between the SOG.R and bilateral ROL, between the MOG.L and IPL.L, between the MOG.R and IPL.L, and between the IOG.R and bilateral insula (INS) (*p* < 0.05, FWE corrected, Figure 2B and Table 3).

### 3.5 PIGD group versus HC group

Compared with the HC group, the PIGD group showed decreased FC between the SOG.L and right inferior frontal gyrus, orbital part (ORBinf.R)/right Heschel gyrus (HES.R)/left anterior cingulate and paracingulate gyri (ACG.L)/bilateral IPL/right superior frontal gyrus, medial (SFGmed.R)/left postcentral gyrus (PoCG.L), between the SOG.R and right hippocampus (HIP.R)/right lenticular nucleus, putamen (PUT.R)/left superior temporal gyrus (STG.L)/right supramarginal gyrus (SMG.R)/DCG.R, between the MOG.L and left cerebellum 4_5 (CER 4_5. L)/ROL.R/DCG.L/left superior parietal gyrus (SPG.L)/PoCG.L, between the MOG.R and CAL.R/right supplementary motor area (SMA.R)/IPL.R/left precentral gyrus (PreCG.L)/right superior frontal gyrus, and dorsolateral (SFGdor.R)/SPG.L, between the IOG.L and CER 4_5. L/STG.L/ROL.R/DCG.L/PreCG.R/PoCG.R, and between the IOG.R and bilateral ROL/SFGmed.L/DCG.L (*p* < 0.05, FWE corrected; Figure 2C and Table 3).

### 3.6 Correlation analysis

**FIGURE 3:**
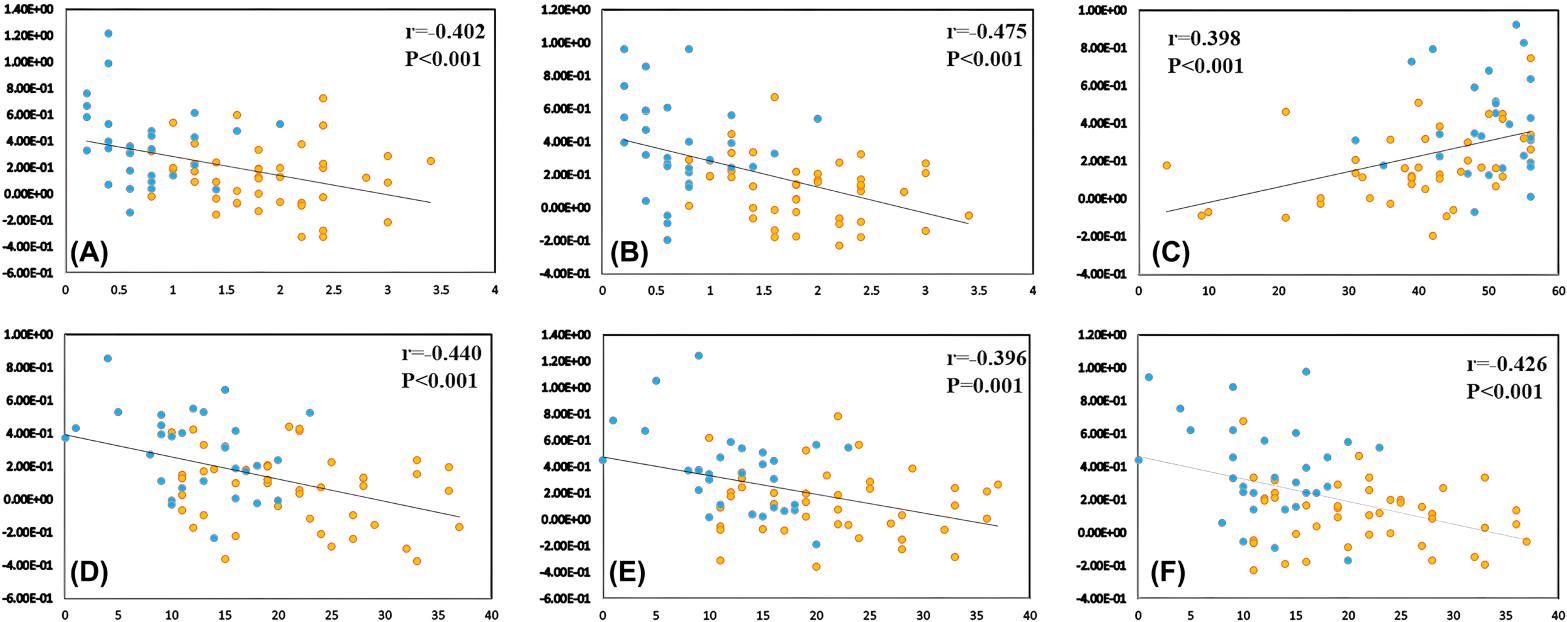
Results of altered FC values and PIGD, MDS‐UPDRSII, BBS scores (*p* < 0.05 FWE corrected). The MOG.R and the PCG.R (A)/ACG.R (B) were negatively correlated with the PIGD scores. The SOG.L and the DCG.R positively correlated with the BBS scores (C). The SOG.L and the IPL.L (D), the MOG.R, and the PCUN.R (E)/ACG.R (F) were negatively correlated with the MDS‐UPDRSII scores. TD patients are represented as blue dots, and PIGD patients are indicated by yellow dots.

In patients with PD, the FC values between MOG.R and PCG.R/ACG.R (*r* = −0.402, *p* < 0.001/−0.475, *p* < 0.001) were negatively correlated with PIGD scores (Figure 3A,B and Table 4). The FC values between the SOG.L and DCG.R (*r =* 0.398, *p* < 0.001) were positively correlated with BBS scores (Figure 3C and Table 4). The FC values between the SOG.L and IPL.L (*r* = −0.440, *p* < 0.001) and between the MOG.R and PCUN.R/ACG.R (*r* = −0.396, *p* = 0.001/−0.426, *p* < 0.001) were negatively correlated with MDS‐UPDRSII scores (Figure 3D–F and Table 4).

**TABLE 4:** Results of correlation of FC values with the clinical scores in PD patients.

Seed ROI	Brain regions (AAL)	Peak value			Voxels	*T* value
		*X*	*Y*	*Z*		
SOG.L	**BBS**					
	DCG.R	6	−30	51	47	3.7318
	**MDS‐UPDRS II**					
	IPL.L	−45	−60	51	67	−5.0805
MOG.R	**MDS‐UPDRS II**					
	ACG.R	9	45	18	56	−4.3419
	PCUN.R	3	−45	18	42	−4.9233
	**PIGD**					
	ACG.R	9	45	18	81	−5.7209
	PCG.R	9	−42	24	56	−4.2765

### 3.7 Classification using ROC analysis

**FIGURE 4:**
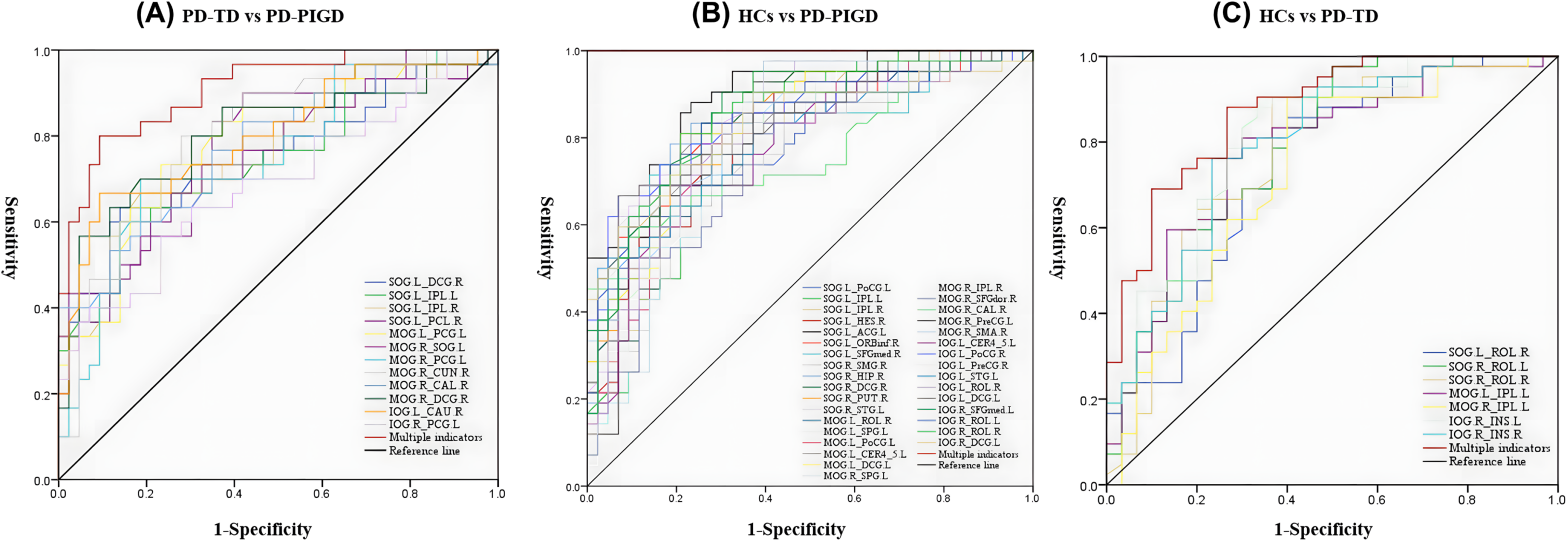
ROC analysis was used to estimate the FC value for distinguishing between TD, PIGD, and HCs. The classification of TD and PIGD (A); the classification of TD and HCs (B); the classification of PIGD and HCs (C).

ROC analysis was used to estimate the FC value to distinguish among the TD, PIGD, and HC groups (*p* < 0.05). An optimal classification model was created by combining multiple parameters with a higher predictive value than that of a single indicator. TD and PIGD multi‐parameter prediction showed an AUC value of 0.910, sensitivity of 80.0%, and specificity of 90.7% (*p* < 0.001; Figure 4A and Table 5). PIGD and HC multi‐parameter prediction exhibited an AUC value of 1, sensitivity of 100%, and specificity of 100% (*p* < 0.001; Figure 4B and Table 5). TD and HC multi‐parameter prediction showed an AUC value of 0.874, a sensitivity of 88.1%, and a specificity of 73.3% (*p* < 0.001; Figure 4C and Table 5).

**TABLE 5:** The AUC values, sensitivity, and specificity in PD patients.

Variables	Sensitivity	Specificity	AUC value	*p* value
PIGD<TD				
SOG.L_DCG.R	63.3%	86.0%	0.755	<0.001
SOG.L_IPL.L	60.0%	86.0%	0.753	<0.001
SOG.L_IPL.R	66.7%	81.4%	0.764	<0.001
SOG.L_PCL.R	83.3%	65.1%	0.761	<0.001
MOG.L_PCG.L	73.3%	76.7%	0.788	<0.001
MOG.R_SOG.L	66.7%	74.4%	0.762	<0.001
MOG.R_PCG.L	70.0%	81.4%	0.753	<0.001
MOG.R_CUN.R	80.0%	72.1%	0.804	<0.001
MOG.R_CAL.R	76.7%	65.1%	0.774	<0.001
MOG.R_DCG.R	56.7%	95.3%	0.802	<0.001
IOG.L_CAU.R	66.7%	90.7%	0.802	<0.001
IOG.R_PCG.L	56.7%	76.7%	0.691	0.006
**Multiple indicators**	**80.0%**	**90.7%**	**0.910**	**<0.001**
PIGD<HC				
SOG.L_PoCG.L	59.5%	90.7%	0.784	<0.001
SOG.L_IPL.L	85.7%	72.1%	0.809	<0.001
SOG.L_IPL.R	81.0%	69.8%	0.802	<0.001
SOG.L_HES.R	88.1%	62.8%	0.803	<0.001
SOG.L_ACG.L	88.1%	76.7%	0.891	<0.001
SOG.L_ORBinf.R	83.3%	69.8%	0.814	<0.001
SOG.L_SFGmed.R	85.7%	72.1%	0.825	<0.001
SOG.R_SMG.R	64.3%	79.1%	0.750	<0.001
SOG.R_HIP.R	83.3%	76.7%	0.837	<0.001
SOG.R_DCG.R	92.9%	62.8%	0.830	<0.001
SOG.R_PUT.R	71.4%	83.7%	0.830	<0.001
SOG.R_STG.L	83.3%	74.4%	0.862	<0.001
MOG.L_ROL.R	85.7%	62.8%	0.805	<0.001
MOG.L_SPG.L	85.7%	69.8%	0.801	<0.001
MOG.L_PoCG.L	81.0%	69.8%	0.790	<0.001
MOG.L_CER4_5.L	81.0%	76.7%	0.825	<0.001
MOG.L_DCG.L	81.0%	79.1%	0.833	<0.001
MOG.R_SPG.L	83.3%	67.4%	0.766	<0.001
MOG.R_IPL.R	83.3%	62.8%	0.780	<0.001
MOG.R_SFGdor.R	81.0%	62.8%	0.759	<0.001
MOG.R_CAL.R	59.5%	88.4%	0.767	<0.001
MOG.R_PreCG.L	66.7%	83.7%	0.788	<0.001
MOG.R_SMA.R	97.6%	60.5%	0.839	<0.001
IOG.L_CER4_5.L	66.7%	83.7%	0.783	<0.001
IOG.L_PoCG.R	66.7%	93.0%	0.842	<0.001
IOG.L_PreCG.R	73.8%	81.4%	0.845	<0.001
IOG.L_STG.L	52.4%	95.3%	0.790	<0.001
IOG.L_ROL.R	78.6%	69.8%	0.780	<0.001
IOG.L_DCG.L	66.7%	93.0%	0.834	<0.001
IOG.R_SFGmed.L	90.5%	69.8%	0.843	<0.001
IOG.R_ROL.L	83.3%	74.4%	0.845	<0.001
IOG.R_ROL.R	81.0%	79.1%	0.860	<0.001
IOG.R_DCG.L	90.5%	62.8%	0.830	<0.001
**Multiple indicators**	**100.0%**	**100.0%**	**1.000**	**<0.001**
TD<HC				
SOG.L_ROL.R	85.7%	60.0%	0.741	0.001
SOG.R_ROL.L	97.6%	50.0%	0.784	<0.001
SOG.R_ROL.R	90.5%	63.3%	0.779	<0.001
MOG.L_IPL.L	81.0%	70.0%	0.778	<0.001
MOG.R_IPL.L	90.5%	60.0%	0.731	0.001
IOG.R_INS.L	88.1%	66.7%	0.814	<0.001
IOG.R_INS.R	76.2%	76.7%	0.796	<0.001
**Multiple indicators**	**88.1%**	**73.3%**	**0.874**	**<0.001**

*Note*: The bold values represent the predictive value of multivaritate indicators.

## 4 DISCUSSION

SOG is responsible for processing complex visual associations and image interpretation. Additionally, it may have a unique function in enhancing the spatiotemporal requirements of gait production.^18^ The MOG is a critical component of the visual cognitive network and essential for integrating visual information. Damage to this region can impair visual and movement perception.^19^ Hyperactivity in the MOG.L and SOG.L might be an underlying compensatory mechanism for PIGD.^13^ The IOG plays a key role in initial eye processing.^20^ Accordingly, we investigated FC changes in the bilateral SOG, bilateral MOG, and bilateral LOG in patients with TD PD, PIGD PD, and HCs. We observed that the subregions of the occipital gyrus, including the SOG.L, MOG.L, MOG.R, IOG.L, and IOG.R, showed significantly different FC changes between patients with TD and PIGD PD. Multiple FC indicators in the occipital gyrus subregions also act as effective biomarkers for detecting TD and PIGD subtypes. In the occipital and cingulate gyri, including SOG.L_DCG.R, MOG.L_PCG.L, MOG.R_PCG.L, MOG.R_DCG.R, and IOG.R_PCG.L, patients with TD PD demonstrated significantly higher FC than patients with PIGD PD. The cingulate gyrus harbors visual signals that facilitate the coordination of eye, head, and body movements.^21^ The dorsal posterior cingulate cortex is implicated in the processing of visuomotor cues.^22^ In addition, our study revealed that the FC values between the SOG.L and DCG.R were positively correlated with BBS scores. The FC values between the MOG.R and ACG.R were negatively correlated with MDS‐UPDRSII scores. The FC values between the MOG.R and ACG.R/PCG.R were negatively correlated with the PIGD scores. Hence, we postulate that patients with TD attain compensation for postural balance and gait through FC between the occipital and cingulate gyri as the disease progresses.

IPL activation correlates with visual feedback pertaining to temporal or spatial movement.^23^ The presence of diminished connectivity in the IPL is linked to disruptions in the neural networks involved in the preparation and initiation of motor functions in PD.^24^ In our study, patients with TD PD demonstrated significantly higher FC than patients with PIGD PD in SOG.L_IPL.L and SOG.L_IPL.R. Additionally, the FC values between SOG.L and IPL.L were negatively correlated with the MDS‐UPDRSII scores, which indirectly supports this perspective. A notable reduction is observed in FC between the SOG.R and PCL.R in individuals with PD‐freezing of gait (FOG) compared with those with non‐FOG, which is related to the control of motor innervation in the lower extremities.^18^ Consistent with this finding, we also observed that patients with PIGD PD had lower FC in SOG.L_PCL.R than patients with TD PD. Microstructural changes in the CAU of patients with PD during the drug‐off state are related to rhythm measurements (rhythm and stride time) of gait.^25^ CAU dopaminergic dysfunction leads to gait impairment, consistent with the correlation between reduced caudate dopamine transporter availability and reduced gait rhythm. Patients with the PIGD PD subtype have additional metabolic reductions in the CAU, compared with those with TD PD subtype.^26^ In this study, the TD PD subtype had a stronger functional link in the IOG.L_CAU.R than the PIGD PD subtype, further verifying that CAU may control gait through the occipital gyrus.

The CUN is related to the pace gait network.^27^ A reduced FC between a branch of the motor cerebellum and the CUN.L is associated with pathophysiological changes related to early visuomotor dysfunction in patients with PD.^28^ The primary visual cortex is located in the calcarine region of the occipital lobe, and the left calcarine GM volume is related to the risk of falling, particularly in patients with PD.^29^ We revealed that patients with PIGD had lower FC in MOG.R_CUN.R, MOG.R_CAL.R, and MOG.R_SOG.L compared to patients with TD. This finding suggests that FC within the visual system contributes to improving gait disorders. We indicate in this study that FC between these brain regions potentially contributes to the differentiation of motor subtypes and the amelioration of PIGD.

In our study, compared with HCs, patients with TD PD showed decreased FC between the SOG.L and ROL.R, between the SOG.R and bilateral ROL, between the MOG.L and IPL.L, between the MOG.R and IPL.L, and between the IOG.R and bilateral INS. Multiple FC indicators between these brain regions also act as effective biomarkers for detecting the HCs and the TD PD subtype. The IPL, ROL, and INS are the cortical regions responsible for sensory and motor functions.^30^, ^31^ The potential FC between the IPL/ROL/INS and the occipital gyrus may contribute to mitigating tremor symptoms in patients with PD. Additionally, in contrast to HCs, patients with PIGD showed decreased FC between the SOG.L and ORBinf.R/HES.R/ACG.L/bilateral IPL/SFGmed.R/PoCG.L, between the SOG.R and HIP.R/PUT.R/STG.L/SMG.R/DCG.R, between the MOG.L and CER 4_5.L/ROL.R/DCG.L/SPG.L/PoCG.L, between the MOG.R and CAL.R/SMA.R/IPL.R/PreCG.L/SFGdor.R/SPG.L, between the IOG.L and CER 4_5.L/STG.L/ROL.R/DCG.L/PreCG.R/PoCG.R, and between the IOG.R and ROL.R/SFGmed.L/ROL.L/DCG.L. Furthermore, multiple FC indicators between these brain regions can be used as effective biomarkers to detect the HCs and the PIGD PD subtype with 100% sensitivity and specificity.

The default mode network (DMN) and dorsal attention network (DAN) are involved in brain areas that are different between PIGD and HCs. A large portion of the DMN is composed of the posterior cingulate cortex, medial prefrontal cortex, inferior parietal cortex, and medial and lateral temporal cortices.^32^ Visuospatial deficits are associated with the loss of anticorrelation patterns in the DMN.^33^ The DAN is composed of the dorsolateral prefrontal cortex, frontal eye fields, SOG, middle temporal motion complex, and superior parietal lobe.^34^ Visual processing requires a DAN, which is known to shape the core system for an emulated action.^35^ The MDS‐UPDRS III motor scores correlate with the DMN, cerebellum, SMA, PUT, and visual (cuneus, lingual, and calcarine) regions.^36^ Motor inhibition and activation are associated with the prefrontal lobe, cingulate gyrus, and supplementary motor cortex.^37^ The TD and PIGD PD subtypes show reduced hippocampal volume compared with HCs, with PIGD showing more dramatic reductions.^38^ The precentral area of the cerebral cortex facilitates the movement of the side limbs. The postcentral area houses the primary sensory cortex, where sensory projections exhibit left/right crossover characteristics.^39^ SMG activity under high‐frequency repetitive transcranial magnetic stimulation is related to the response speed of visuospatial cues.^40^ In this study, we observed that FC in the occipital gyrus and ORBinf/HES/ACG/IPL/SFGmed/PoCG/CER4_5/ROL/DCG/SPG/CAL.R/SMA.R/PreCG.L/SFGdor/STG/PreCG distinguishes between HCs and the PIGD PD subtype, which is significant for a better understanding of PIGD motor symptoms in PD.

This study has some limitations. Initially, the sample size was small, and patients with intermediate PD were not included. Subsequently, the sample size was increased to include patients with intermediate PD. Additionally, as this was a cross‐sectional study, longitudinal observational studies are necessary to clarify whether the FC index used in this study can distinguish between PD motor subtypes.

The PD motor subtypes with differentiated FC of the occipital gyrus and patients with TD PD had significantly higher FC between the SOG.L and DCG.R/PCL.R/bilateral IPL, between the MOG.L and PCG.L, between the MOG.R and SOG.L/CAL.R/DCG.R/PCG.L/CUN.R, between the IOG.L and CAU.R, and between the IOG.R and PCG.L. This shows the importance of FC between the occipital gyrus and the above brain areas in gait regulation and may serve as neural markers to distinguish between patients with TD and PIGD PD. Our findings are valuable for advancing research on motor subtypes of PD and offer a basis for developing tailored treatment strategies for patients with PD.

## CONFLICT OF INTEREST STATEMENT

The authors report no conflicts of interest.

## Supporting information


Supinfo S1.

## Data Availability

Research data are not shared.
