# Supplementary material for: Resting‐state functional connectivity of the occipital cortex in different subtypes of Parkinson's disease
Source: CNS Neurosci Ther. 2024 Aug 26;30(8):e14915. doi: 10.1111/cns.14915 (PMC11347390; doi:10.1111/cns.14915)
Supplement: Supplementary file 1 — Supinfo S1. [file CNS-30-e14915-s001.docx]

The parameters used for obtaining R-fMRI (6 min and 10 s) were as follows: repetition time (TR), 2,000 ms; echo time (TE), 30 ms; flip angle (FA), 90°; field of view (FOV), 240 × 240 mm; matrix, 64 × 64; slice thickness, 3.5 mm; slice gap, 0.5 mm and interleaved slices, 36.

The parameters used to obtain 3D fast spoiled gradient recalled (3DFSPGR) images were as follows: TR, 8.2 ms; TE, 3.2 ms; inversion time, 800 ms; FA, 7°; FOV, 250 × 250 mm; matrix, 256 × 256; and slice thickness, 1 mm.
